# The Triterpenoid CDDO-Methyl Ester Redirects Macrophage Polarization and Reduces Lung Tumor Burden in a Nrf2-Dependent Manner

**DOI:** 10.3390/antiox12010116

**Published:** 2023-01-03

**Authors:** Jessica A. Moerland, Ana S. Leal, Beth Lockwood, Elena Y. Demireva, Huirong Xie, Teresa Krieger-Burke, Karen T. Liby

**Affiliations:** 1 Department of Pharmacology & Toxicology, College of Osteopathic Medicine, Michigan State University, B430 Life Science Building, 1355 Bogue Street, East Lansing, MI 48824, USA; 2 Transgenic and Genome Editing Facility, Institute for Quantitative Health Science & Engineering, Michigan State University, East Lansing, MI 48824, USA; 3 In Vivo Core Facility, Michigan State University, East Lansing, MI 48824, USA

**Keywords:** Nrf2, Keap1, Nrf2 regulators, lung cancer, macrophages, CDDO-Methyl ester, bardoxolone methyl, tumor microenvironment

## Abstract

The NRF2/KEAP1 pathway protects healthy cells from malignant transformation and maintains cellular homeostasis. Up to 30% of human lung tumors gain constitutive NRF2 activity which contributes to cancer cell survival and chemoresistance, but the effects of NRF2 activation in immune cells within the tumor microenvironment are underexplored. Macrophages can promote cancer progression or regression depending on context, and NRF2 activation affects macrophage activity. The NRF2 activator CDDO-Methyl ester (CDDO-Me or bardoxolone methyl) reprogrammed Nrf2 wild-type (WT) tumor-educated bone marrow-derived macrophages (TE-BMDMs) from a tumor-promoting to a tumor-inhibiting phenotype, marked by an increase in M1 markers TNFα, IL-6, and MHC-II and a decrease in the tumor-promoting factors VEGF, CCL2, and CD206. No changes were observed in Nrf2 knockout (KO) TE-BMDMs. CDDO-Me decreased tumor burden (*p* < 0.001) and improved pathological grade (*p* < 0.05) in WT but not Nrf2 KO A/J mice. Tumor burden in Nrf2 KO mice was 4.6-fold higher (*p* < 0.001) than in WT mice, irrespective of treatment. CDDO-Me increased the number of lung-infiltrating macrophages in WT mice but lowered CD206 expression in these cells (*p* < 0.0001). In summary, Nrf2 KO exacerbates lung tumorigenesis in A/J mice, and CDDO-Me promotes an Nrf2-dependent, anti-cancer macrophage phenotype.

## 1. Introduction

Lung cancer is the leading cause of cancer-related mortality in the United States with over 230,000 new diagnoses annually [1]. Lung cancer is not usually diagnosed until the disease has progressed to more advanced stages, which contributes to the 5-year survival rate remaining below 20% [1,2]. The recent incorporation of PD-1/PD-L1 immune checkpoint inhibitors as standard of care for qualifying patients has revolutionized lung cancer therapy for responders [3,4], but less than 20% of patients respond to immunotherapy [5,6]. Although the development of additional treatment strategies is needed for non-responders and refractory patients, the striking effectiveness of these treatments in a subset of patients [5,6,7] highlights the therapeutic potential of immunomodulation for anti-cancer therapies in lung cancer. 

Macrophages are one of the most common immune cells within the lung tumor microenvironment (TME) [8]. These cells exist on a dynamic phenotypic spectrum, which can be either tumor-promoting or tumor-inhibiting [8,9,10] and rapidly change their activity in response to signals within their environment [11,12,13]. Canonically, the macrophage phenotypic spectrum is split between classically activated, pro-inflammatory “M1” macrophages and alternatively activated, anti-inflammatory “M2” macrophages [10]. While this simplistic binary system does not convey the entirety of the complex macrophage phenotypes that exist within the established lung TME, the majority of macrophages within this compartment exhibit characteristics more closely resembling the alternatively activated M2 phenotype [8,13,14]. The ratio of M2:M1 polarized macrophages increases as lung tumor stage progresses [8], and higher infiltration of M2 macrophages in the lung correlates with poor patient prognosis [15,16]. Conversely, increased activity of M1-polarized macrophages correlates with increased patient survival [16]. Therefore, classically activated M1 macrophages can be considered anti-tumorigenic and alternatively activated M2 macrophages can be considered pro-tumorigenic [11]. Modulation of tumor-associated macrophage phenotype has emerged as an attractive therapeutic strategy for the treatment of lung cancer, as skewing these cells away from the M2 phenotype and towards the M1 phenotype can promote anti-tumor effects [17,18,19]. 

Importantly, M1 macrophages rely on cytoprotective pathways to maintain an anti-tumor phenotype and to survive in inflammatory environments [20]. One such cytoprotective pathway is the NRF2 (nuclear factor erythroid factor 2-related factor 2)-KEAP1 (kelch-like ECH-associated protein 1)-ARE (antioxidant response element) pathway. The NRF2 pathway is a master cellular defense mechanism that protects cells from oxidative stress [21] by regulating numerous diverse processes including detoxification, redox-balancing, and metabolism [22]. In healthy cells, NRF2 activation protects against malignant transformation [23,24]. In cancer cells, however, the cytoprotective properties of NRF2 pathway activation contribute to increased tumor cell survival and resistance to anti-cancer therapies [24,25]. Because of the hypoxic nature of tumors, cancer cells have elevated levels of NRF2 signaling well above the basal level found in normal cells [26,27], and up to 30% of lung cancers acquire a mutation within the tumor cells which results in constitutive NRF2 pathway activation [28]. The majority of these alterations are loss-of-function mutations in KEAP1, the negative regulator of the NRF2 protein [29]. The summation of these characteristics results in tumor cells having elevated NRF2 activity levels, regardless of mutational status. 

However, the effects of NRF2 pathway activation in the immune cells within the TME are not well understood. Initial observations on the function of Nrf2 in macrophages suggested anti-inflammatory effects [30]; however, these studies were not repeated in the context of cancer. Further studies revealed that myeloid-specific deletion of Nrf2 promoted tumorigenesis [31]; conversely, tumor burden decreased when Nrf2 was selectively activated in the TME [32]. However, these studies did not differentiate between the different immune cell populations within the microenvironment. Moreover, whole-body Nrf2 knockout in Balb/c mice resulted in an increased lung tumor burden and a unique immune signature within the lungs, which was characterized by elevated levels of tumor-promoting macrophages [33]. More recently, potential anti-tumor activity of Nrf2-activated macrophages has been proposed [34,35], but this mechanism remains underexplored.

Synthetic oleanane triterpenoids are among the most potent known pharmacological activators of the NRF2 pathway [36,37] and function by modifying cysteine residues on the negative regulator KEAP1 to prevent NRF2 degradation and promote pathway activation [36]. The derivative CDDO-Methyl ester (CDDO-Me or bardoxolone methyl) reduced tumor burden by more than 90% in a preclinical mouse model of lung cancer [38,39] and has been tested in clinical trials for treatment of chronic kidney disease [40] and pulmonary arterial hypertension [41]. However, the mechanism of this anti-tumor activity and the specific effects of CDDO-Me on tumor-associated macrophages have not been characterized. Because of the cytoprotective effects of NRF2 activation in tumor cells, some investigators have raised concerns whether the clinical use of NRF2 activators could lead to disease progression and drug resistance [42,43]. However, multiple preclinical studies suggest potent anti-tumor activity for these small molecules, possibly by regulating cells of the microenvironment rather than tumor cells [38,39,44]. Here, we investigate if CDDO-Me reprograms tumor-associated macrophages in a Nrf2-dependent manner to create an anti-tumor immune microenvironment in a carcinogen-induced model of preclinical lung cancer.

## 2. Methods

### 2.1. Generation of Nrf2 KO Mice

Nrf2 KO mice were created on an A/J genetic background by CRISPR-Cas9 editing of the Nrf2 locus ENSMUSG00000015839 (Accession # NM_010902.5). Two guide RNAs were used to target the gene, N_(20)_ *PAM*: 5′-AGCGTGGCTGGGGATATCCA *GGG*-3′ in exon 4, and 5′-GTAAATGAGTGATTGTCCTA *TGG*-3′ downstream of exon 5. Zygotes from A/J mice (Jackson Laboratory, stock # 000646) were electroporated with 100 ng/uL ribonucleoprotein complexes comprised of equimolar amounts of WT NLS-Cas9 protein, and pre-hybridized synthetic crRNA and tracrRNA (Integrated DNA Technologies, Inc., Coralville, IA, USA). Electroporation was performed at 30 V with 2 pulses 1 s apart with a Gene Editor electroporator (BEX CO., LTD, Tokyo, Japan) as previously described [45]. Edited embryos were transferred into pseudopregnant recipients using standard procedures. Resulting founder litters were analyzed by PCR and Sanger sequencing for editing of the target site. One positive founder was identified with a 16 bp deletion at the beginning of exon 4. This resulted in a frameshift following A137 with the first premature termination codon (PTC) occurring at amino acid position 161. A second indel on the same allele was identified at the second gRNA cut site downstream of exon 5—a 1 bp deletion which does not affect the coding sequence. Off-target analysis revealed no significant off-target hits were predicted on the same chromosome or in coding regions. A stable Nrf2 KO line was established by breeding the founder with wildtype A/J mice for several generations. 

### 2.2. Genotyping of Nrf2 KO Mice

Tail biopsies were lysed with proteinase K and used for PCR. For genotyping, the region around the exon 4 indel was amplified with primers F 5′-GGAGTCACTGGGAGGAGGAA-3′ and R 5′-CGCAGAAAACAGCACACTCC-3′ and the difference between the WT and mutant allele was distinguished either by Sanger sequencing, T7 Endonuclease I assay (New England BioLabs, Inc., Ipswich, MA, USA), or the loss of an EcoRV site naturally occurring on the WT allele but disrupted by the indel on the edited allele.

### 2.3. Cell Culture

LL2 mouse lung cancer cells (ATCC) and VC-1 cells were cultured in DMEM supplemented with 5% FBS and 1% Pen/Strep at 37 °C and 5% CO_2_. Cells were switched to RPMI1640 with 5% FBS and 1% Pen/Strep 48 h prior to conditioned media collection. For conditioned media collection, cells were plated at ~30% confluency in RPMI1640 supplemented with 1% FBS and allowed to grow for 24 h, after which media was collected while the cells were in the exponential growth phase. Conditioned media was spun down at 1000 RPM for 5 min to pellet any remaining cell debris, then either added to macrophage cultures immediately or frozen at −20 °C for <1 week. Cells were not used after 10 passages. 

### 2.4. Bone Marrow-Derived Macrophage (BMDM) Isolation

Femurs and tibias were harvested from female A/J WT or Nrf2 KO mice and bone marrow flushed into RPMI1640 media containing 20 ng/mL M-CSF. 500 K cells were plated onto 6-well plates and incubated at 37 °C and 5% CO_2_ for five days. On the 6th day, media was aspirated and cells were washed three times with PBS. Media was replaced with RPMI1640 containing a reduced dose of 10 ng/mL M-CSF (BioLegend #576406, San Diego, CA, USA). Cells were stimulated with either 10 ng/mL LPS (Sigma, St. Louis, MO, USA), 10 ng/mL IFN-γ (R&D Systems #485-MI, Minneapolis, MI, USA), 10 ng/mL IL-4 (BioLegend #574304), or 50–75% conditioned media from LL2 cells and treated with either DMSO vehicle control or CDDO-Me at 100 nM for 24 h. 

### 2.5. Quantitative Real-Time PCR

Total RNA was isolated with TRIzol (Invitrogen, Waltham, MA, USA), and 2 µg RNA was used to synthesize cDNA using the SuperScript III reverse transcriptase kit (Invitrogen) and optimized PCR conditions: 10 min 25 °C; 2 h 37 °C; 5 min 85 °C; ∞ 4 °C. Primers were either ordered from IDT or Qiagen (Table S1) and an optimized qPCR protocol (2 min 50 °C; 10 min 95 °C; 15 s 95 °C; 1 min 60 °C; repeat steps 3–4 40×; 15 s 95 °C; 1 min 60 °C) was run using the QuantStudio 7 Flex Real-Time PCR system (ThermoFisher, Waltham, MA, USA). Fold change in mRNA expression was quantified using the ddCT method normalized to WT unstimulated (M-CSF only) control. 

### 2.6. Treatment of Lung Adenocarcinomas In Vivo

All experiments were performed in accordance with AAALAC-accredited Standards for the Management of Laboratory Animals at Michigan State University (MSU). All protocols were approved by the Institutional Animal Care and Use Committee (IACUC, protocol 202100188) at MSU. At 7 and 8 weeks of age, male and female WT A/J mice (originally obtained from Jackson Laboratories and bred in-house) and Nrf2 KO A/J mice (described above) were injected i.p. with vinyl carbamate (Toronto Research Chemicals, 0.32 mg/mouse). Mice were fed a semi-synthetic diet (AIN-93G BioServ, Raritan Township, NJ, USA), beginning one week before initiation with vinyl carbamate. The mice were then randomized into groups and fed control or treatment diets (12.5–50 mg CDDO-Me/kg AIN-93G diet or ~3.1–12.5 mg/kg body weight) for 16 weeks, starting one week after the final injection of vinyl carbamate. CDDO-Me was dissolved in a vehicle of 12.5 mL ethanol and 37.5 mL Neobee oil (Spectrum Chemical, New Brunswick, NJ, USA) per kg AIN-93G diet. After 15 weeks on treatment diet, lungs were imaged using ultrasound as previously described [46]. After 16 weeks on treatment diet, blood and lungs were harvested. Lungs were inflated with PBS and grossly visible lung tumors on the surface of the lungs were counted. The left lung was fixed in neutral-buffered formalin (NBF), embedded in paraffin, step-sectioned (2 sections/lung, 800 microns apart) and either stained with Hematoxylin and Eosin or analyzed by IF. Samples were blinded as to group and randomized before the number, size, and histopathology of tumors on the slides were evaluated as described [39]. The right lobes of the lung were divided into halves. One half was analyzed by flow cytometry and the other half flash frozen and stored at −80 °C.

### 2.7. Flow Cytometry

The same two lobes of the right lung were harvested from each A/J mouse (n = 7–9/group) and incubated in digestion media consisting of collagenase (300 U/mL, Sigma) and DNAse (2 U/mL, Calbiochem, San Diego, CA, USA) for 30 min at 37 °C with stirring. Samples were then passed through a 40 µm cell strainer (ThermoFisher, Waltham, MA, USA), and red blood cells eliminated with lysis solution. Single cells were resuspended in a solution of Brilliant Violet buffer (BD Bioscience, Franklin Lakes, NJ, USA) and stained for 30 min at 4 °C with an optimized antibody panel [47] and 5 μg/mL anti-mouse CD16/CD32 antibody (Biolegend) to reduce antibody binding to Fc receptors. Cells were analyzed using a Cytek Aurora spectral flow cytometer equipped with 5 lasers (UV 355 nm, violet 405 nm, blue 488 nm, yellow-green 561 nm, and red 640 nm) and FlowJo x.10.0.7r2 software (Tree Star, Ashland, OR, USA). The gating strategy used was adapted from a published protocol [47].

### 2.8. ELISAs

Culture supernatant was harvested from BMDMs, centrifuged at 1000 RPM for 5 min, and supernatant was removed from the pellet and frozen at −80 °C. Protein levels were quantified using sandwich ELISAs for mouse TNFα (Invitrogen, # 88-7324-22), IL-6 (R&D Systems, # SM6000B), VEGF (R&D Systems, # SMMV00), and CCL2 (R&D Systems, # SMJE00B).

### 2.9. Immunofluorescent Staining

Paraffin blocks of the left lobe of the lung were sectioned, immunostained with antibodies against CD206 (1:200, Abcam #ab64693, Cambridge, UK) and NQO1 (1:100, ThermoFisher #PA5-115666) and visualized with anti-rabbit Alexa Fluor Plus 647 IgG (ThermoFisher #A32733TR). Sections were counterstained with DAPI and coverslips mounted with ProLong Gold (Invitrogen #P36941). Slides were visualized using an Eclipse Ti2-E Nikon microscope and images were analyzed and quantified using FIJI software.

### 2.10. Western Blotting

Lungs from tumor-bearing WT and Nrf2 KO A/J mice were homogenized in EBC buffer containing protease inhibitors (PMSF, aprotinin, and leupeptin) and 10% NP-40 and incubated on ice for 40 min with agitation and vortexing and sonication every 10 min. Protein concentrations were determined by the BCA assay (Sigma-Aldrich, St. Louis, MO, USA), and concentrations were normalized in resolving SDS-PAGE buffer. Following electrophoresis (100 V) through 10% polyacrylamide gel, protein was transferred onto a nitrocellulose membrane (100 V for 2.5 h) and probed for NQO1 (1:1000, 5% BSA, Invitrogen #PA5-115666), β-actin (1:1000, 5% BSA, Sigma-Aldrich #A-5441) and rabbit and mouse fluorescent secondary antibodies (1:1000, 5% BSA, LI-CORE, #926-32213 and #926-32212, respectively). Membranes were visualized using a LI-CORE Clx and quantified using Image Studio software. 

### 2.11. Statistical Analysis

All statistics for *in vitro* studies were done using GraphPad Prism 9 software. Two-way ANOVA followed by Tukey HSD was used for analysis of mRNA, protein, and cell surface marker expression. For in vivo studies, Student *t* test was used to compare two groups, and 2-way ANOVA followed by Tukey HSD was used when comparing >2 groups. Tumor histopathology proportions were compared using the z-test. Alpha was set to α = 0.05.

## 3. Results

### 3.1. Generation of Constitutive Nrf2 KO A/J Mice

**Figure 1:**
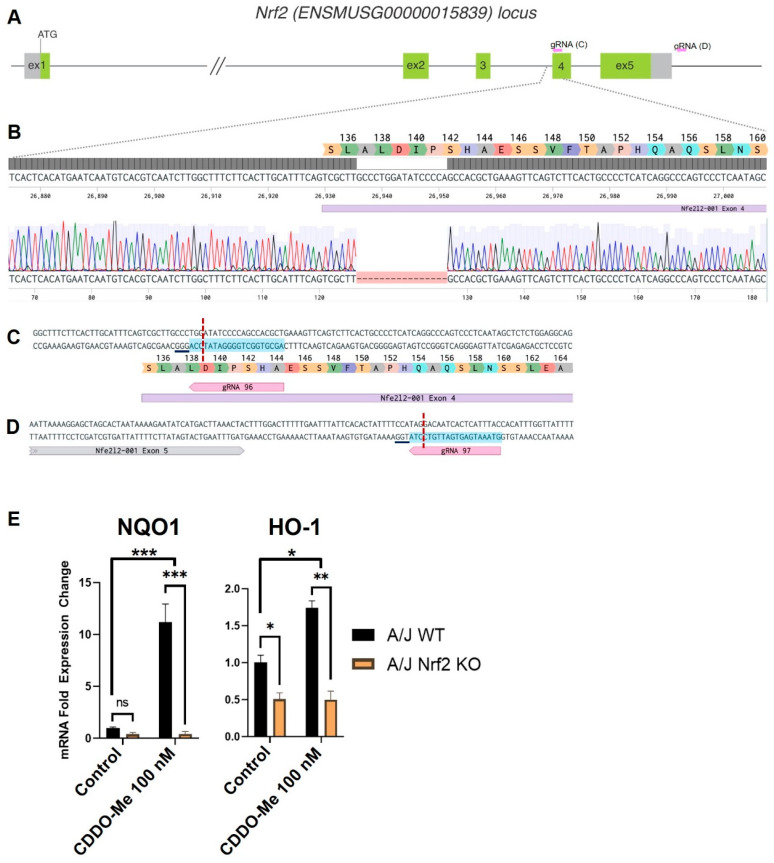
Targeting of the Nrf2 locus in A/J mice. (**A**) Schematic of the Nrf2 (*Nfe2l2*) mouse locus showing relative positions of exons 1–5. Non-coding regions of exons indicated in gray with start site (ATG) located in exon 1. Exon length not to scale. Relative location of gRNAs indicated in pink arrows and expanded in C and D. (**B**) Magnified view of the target region. Benchling sequence alignment between the WT reference sequence of Nrf2 (*Nfe2l2*) and a raw sequence chromatogram from a F2 Nrf2 KO animal, showing a 16 bp deletion at the beginning of exon 4 (purple bar). Corresponding translation of exon 4 is included as a reference. (**C**,**D**) detailed sequence and position of gRNA targets in exon 4 and downstream of exon 5, respectively. Protospacer—blue highlight, DSB—red dotted line, underline—PAM. (**E**) Differential mRNA expression of Nrf2 target genes NQO1 and *HMOX-1* in bone marrow derived macrophages isolated from WT and Nrf2 KO A/J mice treated with vehicle or CDDO-Me for 24 h. (**E**) is representative of 3 independent repeat experiments, each with 3 technical replicates. Two-way ANOVA followed by Tukey HSD. ns = not significant, * *p* < 0.05, ** *p* < 0.01, *** *p* < 0.001.

To test the necessity of Nrf2 activation for the anti-tumor effects of CDDO-Me, we generated a new Nrf2 knockout (KO) mouse on the A/J background. This strain was selected because A/J mice are highly susceptible to carcinogens and subsequent lung carcinogenesis [48], and previous studies evaluating the anti-tumor activity of CDDO-Me were completed in WT mice on this genetic background. CRISPR-Cas9 editing of the mouse Nrf2 (ENSMUSG00000015839) locus (Figure 1A–D) [49], resulted in a 16 bp deletion at the beginning of exon 4 (Figure 1B). This deletion results in a frameshift after codon A137, and the mutant mRNA is predicted to be degraded by the nonsense-mediated mRNA decay (NMD) mechanism [50]. In the event that the mutant message escapes NMD degradation, it is predicted to produce a nonfunctional protein, truncated at residue 160, which cannot activate target gene transcription because of disruption of the nuclear export signal domain [51]. To confirm the functional disruption of Nrf2 in the newly created A/J Nrf2 KO mice, bone marrow-derived macrophages (BMDMs) were isolated from A/J WT and Nrf2 KO mice and treated with vehicle or the Nrf2 activator CDDO-Me. As expected, mRNA expression of the Nrf2 target genes NQO1 and *HMOX-1* was significantly higher (*p* < 0.001 and *p* < 0.01, respectively) in WT BMDMs treated with CDDO-Me (Figure 1E), but no increase of target gene expression was detected in Nrf2 KO BMDMs treated with CDDO-Me. Additionally, basal levels *of HMOX-1* were lower in KO cells compared to WT, further supporting the lack of functional Nrf2. These findings confirm that the Nrf2 protein is not functional in Nrf2 KO A/J mice.

### 3.2. CDDO-Me Has Anti-Inflammatory Nrf2-Dependent Effects in BMDMs Stimulated with LPS or IFN-γ

**Figure 2:**
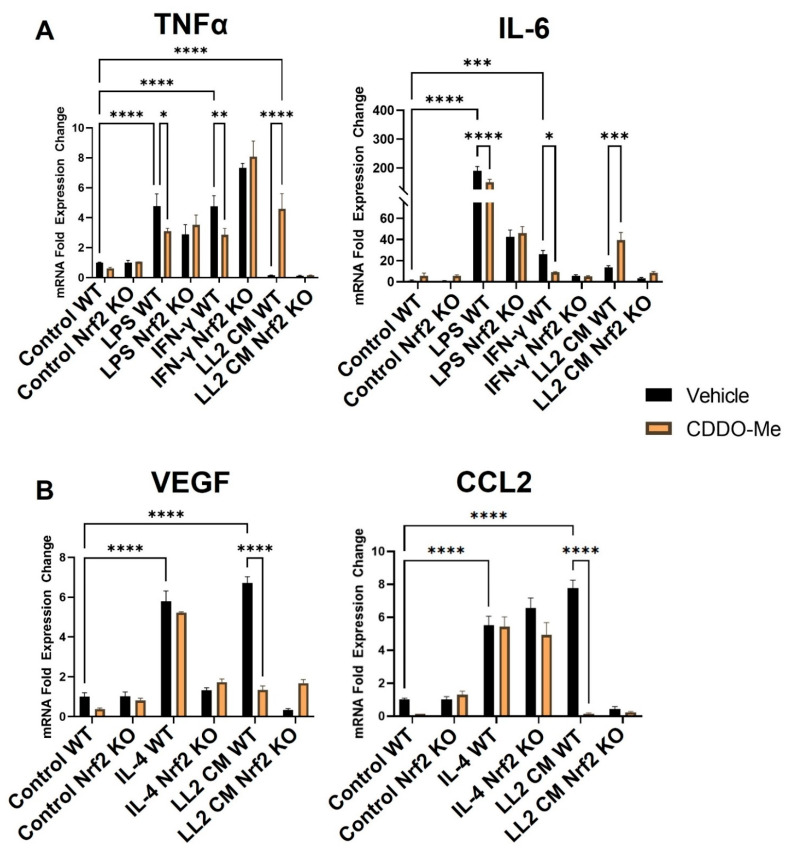
CDDO-Me has opposing Nrf2-dependent effects on macrophage polarization when stimulated with LPS or IFNγ vs. conditioned media from lung cancer cells. Bone marrow-derived monocytes were isolated from A/J WT and Nrf2 KO mice and differentiated with M-CSF for 5 days. Bone marrow-derived macrophages were stimulated with 10 ng/mL LPS or 10 ng/mL IFN-γ to induce a M1 (**A**) phenotype, 10 ng/mL IL-4 to induce a M2 (**B**) phenotype, or conditioned media (CM) from LL2 lung cancer cells to induce a tumor-educated (**A**,**B**) phenotype. BMDMs were then treated with vehicle (black bars) or 100 nM CDDO-Me (orange bars) for 24 h and mRNA expression was analyzed by qPCR. Representative of 3 independent repeat experiments, each containing 3 technical replicates. Two-way ANOVA followed by Tukey HSD. ns = not significant, * p < 0.05, ** *p* < 0.01, *** *p* < 0.001, **** *p* < 0.0001.

To validate the responsiveness of macrophages in this model, monocytes were isolated from Nrf2 WT and KO mice and treated with M-CSF for 5 days to induce macrophage differentiation. Consistent with previous studies [52,53], treatment with LPS (10 ng/mL) and IFN-γ (10 ng/mL) for 24 h (Figure 2A) increased mRNA expression of the M1 macrophage markers TNFα ~5-fold (*p* < 0.0001) and IL-6 ~200- and ~40-fold, respectively (*p* < 0.0001). IL-4 (10 ng/mL) increased expression of the M2 macrophage markers VEGF (*p* < 0.0001) and CCL2 (*p* < 0.0001) by ~6-fold (Figure 2B). To ensure the rigor of these contrasting phenotypic changes, BMDM polarization was further characterized at the levels of secreted protein and cell surface markers. BMDMs were isolated and stimulated as described, and culture supernatant was collected to detect secreted protein expression by ELISA. Consistent with transcriptional changes, LPS and IFN-γ increased TNFα and IL-6 protein secretion to 400–450 pg/mL (*p* < 0.0001), and IL-4 increased VEGF and CCL2 protein secretion to 60 and 250 pg/mL, respectively (*p* < 0.05) (Figure S1A,B). Additionally, BMDMs were isolated as described and treated for 48 h to evaluate surface marker expression. The M1 marker MHC-II increased by ~50% in BMDMs stimulated with LPS and IFN-γ (*p* < 0.0001) compared to an unstimulated control, and the M2 marker CD206 was increased by a similar magnitude in BMDMs stimulated with IL-4 (*p* < 0.01) (Figure S2A,B).

Treatment with 100 nM CDDO-Me for 24 h decreased TNFα and IL-6 mRNA (Figure 2A) in WT M1 BMDMs stimulated with LPS (*p* < 0.05 and *p* < 0.0001) and IFN-γ (*p* < 0.01 and *p* < 0.05). This observation is consistent with the reported anti-inflammatory effects in macrophages following Nrf2 activation [30]. CDDO-Me also decreased TNFα and IL-6 secreted protein in WT LPS (*p* < 0.05 and *p* < 0.01, respectively) and IFN-γ (*p* < 0.05)-stimulated BMDMs (Figure S1A). In Nrf2 KO BMDMs, however, CDDO-Me treatment did not change expression of any of these cytokines, indicating a dependence on Nrf2 activation (Figure 2A and Figure S3A). CDDO-Me had no effect on either WT or Nrf2 KO M2 macrophages stimulated with IL-4 at the transcriptional level (Figure 2B) or the level of secreted protein (Figure S1B), regardless of stimulation. 

### 3.3. Conditioned Media from Lung Cancer Cells Reverses the Anti-Inflammatory Effect of CDDO-Me and Promotes a Nrf2-Dependent Anti-Tumor Phenotypic Profile in Tumor-Educated BMDMs

**Figure 3:**
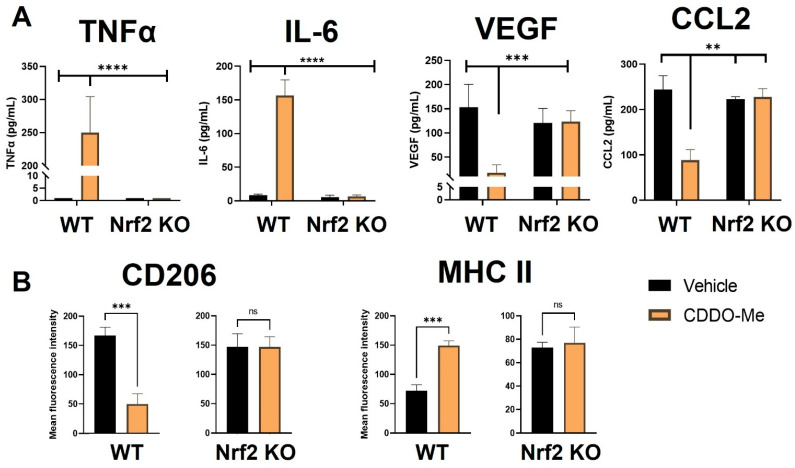
CDDO-Me induces an anti-tumor macrophage phenotype in tumor-educated bone marrow-derived macrophages in a Nrf2-dependent manner. (**A**) Bone marrow-derived monocytes were isolated from A/J WT and Nrf2 KO mice and differentiated into tumor-educated BMDMs as described in Figure 2. BMDMs were treated with vehicle (black bars) or 100 nM CDDO-Me (orange bars) for 24 (**A**) or 48 (**B**) h. TNFα, IL-6, VEGF, and CCL2 protein expression was analyzed by ELISAs in (**A**). Mean fluorescence intensity of CD206 and MHC-II cell surface markers was evaluated by flow cytometry (**B**). Representative of 3 independent repeat experiments. Two-way ANOVA followed by Tukey HSD (**A**) or unpaired T test (**B**). ns = not significant, ** *p* < 0.01, *** *p* < 0.001, **** *p* < 0.0001.

Notably, BMDMs were also cultured in conditioned media from LL2 (Figure 2A) and VC-1 (Figure S3A) murine lung cancer cell lines to induce a lung tumor-educated phenotype. Conditioned media from both lung cancer cell lines increased (*p* < 0.0001) VEGF and CCL2 mRNA expression by 6- and 8-fold, respectively, indicating successful polarization to a tumor-educated phenotype [54,55]. Conditioned media from LL2 cells also increased VEGF (*p* < 0.0001) and CCL2 (*p* < 0.01) secreted protein to 150–250 ng/mL (Figure S1B). In striking contrast to BMDMs stimulated with LPS and IFN-γ, CDDO-Me increased TNFα (*p* < 0.0001) and IL-6 (*p* < 0.001) mRNA ~4-fold in WT tumor-educated BMDMs (Figure 2A). These results were complemented by a decrease in mRNA expression of the tumor-promoting factors VEGF (*p* < 0.0001) and CCL2 (*p* < 0.0001) to baseline levels in response to CDDO-Me treatment in tumor-educated BMDMs (Figure 2B). Importantly, similar trends in all four phenotypic markers were observed at the transcriptional level in BMDMs educated with conditioned media from LL2 (Figure 2) and VC-1 cells (Figure S3). CDDO-Me also highly increased (*p* < 0.0001) TNFα and IL-6 secreted protein to ~150–250 ng/mL but decreased VEGF (*p* < 0.001) and CCL2 (*p* < 0.01) protein by ~60–90% in WT BMDMs educated with conditioned media from LL2 cells (Figure 3A). At the cell surface marker level, CDDO-Me decreased (*p* < 0.001) CD206 MFI by ~60% but increased (*p* < 0.001) MHC-II MFI ~3-fold in LL2-educated BMDMs (Figure 3B). No differences in any of these phenotypic markers were observed in Nrf2 KO tumor-educated BMDMs treated with CDDO-Me, indicating that the observed effects were Nrf2 dependent. 

The Nrf2-dependent increase in expression of the M1 macrophage markers TNFα, IL-6, and MHC-II and the decrease in the M2 macrophage markers VEGF, CCL2, and CD206 at the transcriptional, protein, and surface marker level in macrophages stimulated with conditioned media from lung cancer cells suggest that CDDO-Me redirects the polarization of lung tumor-educated macrophages to an anti-tumor phenotype through the activation of Nrf2. To fully model the dynamic interactions between Nrf2, macrophages and other cells within the lung TME, CDDO-Me treatment effects were tested in vivo.

### 3.4. Nrf2 KO Increases Lung Tumor Burden and CDDO-Me Decreases Lung Tumor Burden in A/J Mice in a Nrf2-Dependent Manner

**Figure 4:**
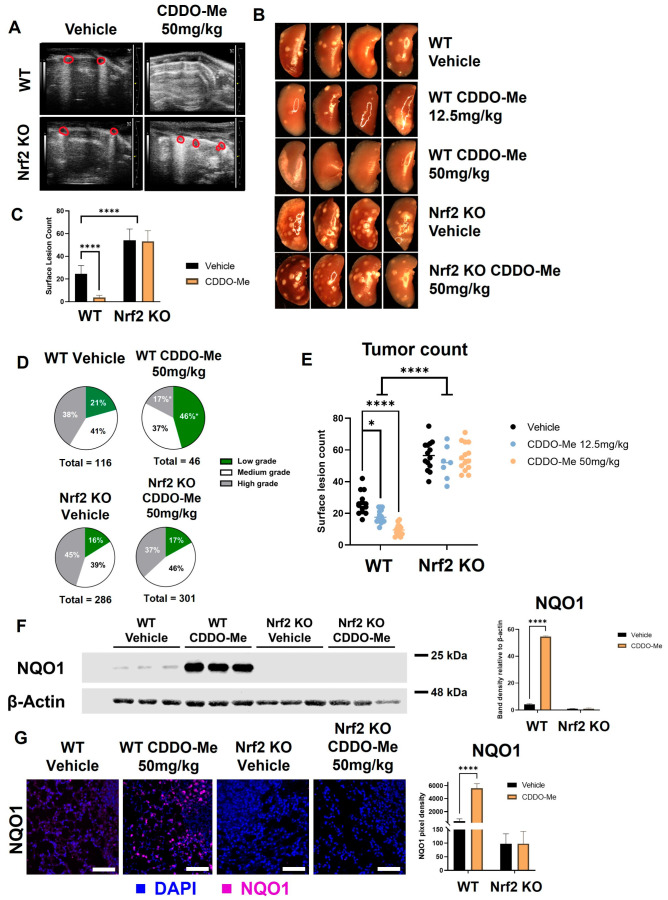
CDDO-Me decreases lung tumor burden in a Nrf2-dependent manner. WT and Nrf2 KO A/J mice were challenged with vinyl carbamate to induce lung tumors and then treated with vehicle control or CDDO-Me (12.5–50 mg/kg of diet) for 16 weeks. (**A**) Ultrasound of WT and Nrf2 KO mice after 15 weeks of treatment. Lung tumors are outlined in red. (**B**) Representative images (8×) of the left lung. Quantification of total surface tumors (**C**,**E**). Histopathological grades on tumor sections (**D**). Western blotting (**F**) and immunofluorescent staining (**G**) of NQO1 in the lungs of mice. Scale bar = 60 microns. Quantification of panels (**F**,**G**) were done using LI-COR Image Studio software (**F**) or FIJI software (**G**); NQO1 is normalized to β-actin in panel **F**. Representative images of n = 5 (**A**), n = 5–12 (**B**–**E**), or n = 3 (**F**–**G**) mice. Two-way ANOVA followed by Tukey HSD (**C**,**E**–**G**) or z-test (**D**) for comparisons of proportions between treatment groups (e.g., low grade WT control vs. low grade WT CDDO-Me) * *p* < 0.05; **** *p* < 0.0001.

Lung tumorigenesis was initiated with vinyl carbamate in WT and Nrf2 KO A/J mice, as previously described [56]. Mice were fed either a vehicle control diet or a diet containing CDDO-Me at 50 mg/kg of diet (~12.5 mg/kg of body weight). Obvious differences in tumor burden between the control and CDDO-Me treatment groups in WT mice were detected by ultrasound [46] after 15 weeks on diet (Figure 4A) in a small cohort of mice. Therefore, a total of 16 weeks on treatment diet was chosen as the study endpoint. Mice were weighed weekly to monitor toxicity, and no significant differences in weight were observed between groups at study endpoint (Table S2). 

Immediately after euthanasia, lungs were inflated for quantification of surface tumors. The differences between treatment groups were quite striking and immediately detectable when lungs were removed. These impressive differences can be appreciated in representative gross images taken of the left lung (Figure 4B) and upon quantification of surface lesions (Figure 4C). Interestingly, Nrf2 KO mice had more surface tumors (*p* < 0.0001), regardless of treatment, compared to WT mice treated with vehicle control. Surface lesion counts were lower in WT mice treated with CDDO-Me (*p* < 0.0001) than in controls, with an average of 4.4 ± 0.9 and 26.9 ± 1.9 tumors per mouse, respectively (Table 1). In Nrf2 KO mice, however, CDDO-Me treatment had no impact on the average number of surface tumors.

After quantification of surface tumors, the left lung was fixed and step-sectioned for histopathological analysis. All analyses of histological sections were done in a randomized, double-blind fashion by two independent researchers. Number and size of tumors per slide were evaluated, and tumors were graded for histological and nuclear severity as previously described [46]. The marked increase in surface tumor count in Nrf2 KO mice compared to WT mice was reflected in the histological sections. Average number, size, and burden of vehicle-treated mice were significantly higher in Nrf2 KO animals (247%, 189%, and 467% of the number, size, and burden of vehicle-treated WT mice, respectively) (Table 1). Treatment with CDDO-Me in WT mice effectively reduced the average number of lung tumors per slide by 62% (*p* < 0.01), tumor size by 83% (*p* < 0.001), and tumor burden by 95% (*p* < 0.001) compared to the vehicle-treated WT control group (Table 1). The proportion of high-grade (more severe) tumors was only 17% in the CDDO-Me group compared to 41% in the control WT group (*p* < 0.05), and the percentage of the lowest grade (least severe) tumors was more than two-fold (*p* < 0.05) higher in the WT mice treated with CDDO-Me (46%) vs. WT controls (21%; Figure 4D and Table 1). In contrast to the striking efficacy in the WT mice treated with CDDO-Me, no changes in average tumor number, size, burden, or histopathology were observed in any of the Nrf2 KO groups.

### 3.5. Sex-Dependent Differences in Lung Carcinogenesis in A/J Mice

While sex-related differences have been reported in benzo[a]pyrene-induced A/J tumorigenesis [57], sex differences in A/J mice injected with vinyl carbamate have not been explored. In a similar trend as observed in Table 1, where data from both sexes were combined, the average number of surface tumors, tumor number, and burden were higher in both male and female Nrf2 KO mice compared to their WT controls (Table S3). However, male Nrf2 KO mice had a 2-fold increase (*p* < 0.05) in average tumor burden (4.05 ± 0.6 mm^3^ in males compared to 1.9 ± 0.3 mm^3^ in females) and an overall trend toward increased severity in tumor histopathology compared to female Nrf2 KO mice (Table S3). Similar Nrf2-dependent efficacy with CDDO-Me was observed in the lungs of both male and female WT mice, although these differences were not as pronounced in the males as in the female mice. 

### 3.6. CDDO-Me Activates the Nrf2 Pathway In Vivo

To confirm Nrf2 pathway activation in WT mice treated with CDDO-Me, levels of NQO1 protein in the lungs of treated mice were quantified by Western blot (Figure 4F). A faint band of NQO1 was detectable in WT A/J mice treated with vehicle control, illustrating basal Nrf2 activity in the carcinogen-challenged WT mouse lung [58]. In WT mice treated with 50 mg CDDO-Me/kg diet, the NQO1 band intensity increased over 50-fold (*p* < 0.0001), indicating activation of Nrf2 by CDDO-Me in the lungs. Critically, no detectable NQO1 band was observed in Nrf2 KO A/J mice, regardless of treatment. NQO1 immunofluorescent staining was also approximately 5-fold (*p* < 0.0001) higher in the lungs of WT mice treated with CDDO-Me vs. control but was absent in the lungs of all Nrf2 KO groups (Figure 4G). 

To test the dose-dependency of the decrease in tumor burden observed with CDDO-Me treatment, a smaller cohort of mice was fed either vehicle control diet or 12.5 mg CDDO-Me/kg diet (~3.125 mg/kg of body weight) for 16 weeks. Treatment with this lower dose decreased average surface tumor count from 25.5 ± 1.8 in the control group to 14.9 ± 0.6 tumors per mouse (*p* < 0.05), but this magnitude of this decrease was lower than the 50 mg CDDO-Me/kg of die) dose (*p* < 0.0001), which decreased surface tumor count 6-fold (from 26.9 ± 1.9 in the control group to 4.4 ± 0.9 tumors per mouse) (Table 1, Figure 4E). This dose-dependent effect can be appreciated in gross images take of the left lung of these mice, as shown in Figure 4B. No change was observed in the number of surface tumors in Nrf2 KO mice treated with CDDO-Me at this lower dose, again confirming Nrf2 dependency (Figure S4). The lower dose of 12.5 mg/kg CDDO-Me decreased the average tumor number, size, and burden on sections, which were 38%, 42.3%, and 30.9% of the WT controls, respectively (Table S4). While these changes were significant (*p* < 0.05), this lower dose was not as effective as the higher 50 mg/kg dose of CDDO-Me, which reduced tumor number, size, and burden to 38%, 17%, and 8% of the control group, respectively (Table 1). The lower dose was not sufficient to significantly improve tumor histopathological grades in WT mice, although there was a trend toward less severe tumors in these lungs (Table S4). 

### 3.7. CDDO-Me Increases Infiltration and Modulates Polarization of Lung Macrophages in a Nrf2- and Context-Dependent Manner

**Figure 5:**
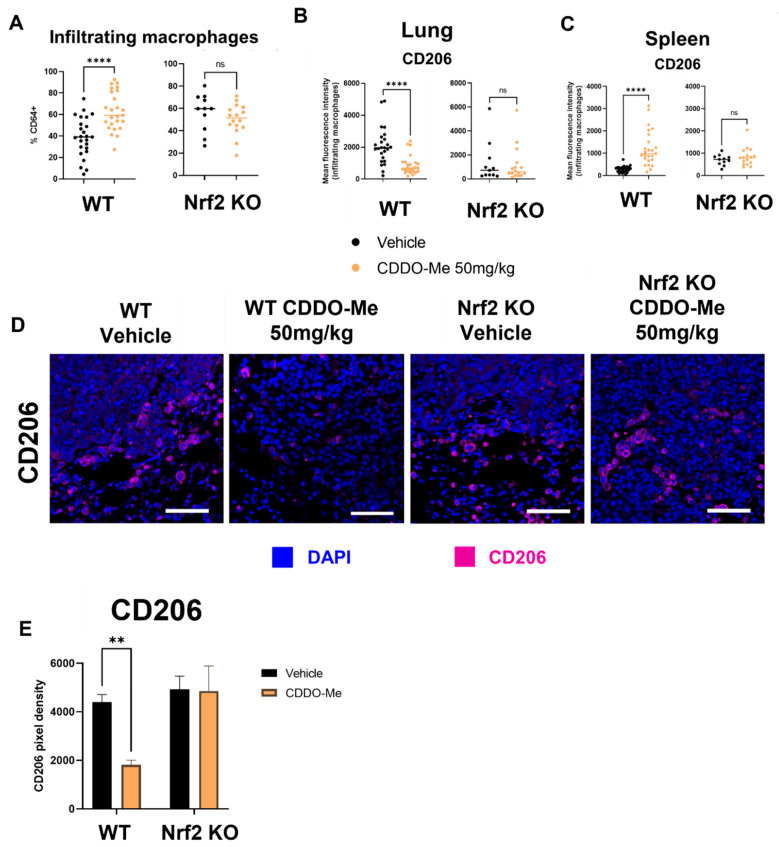
CDDO-Me modulates macrophage infiltration and polarization in the lungs of A/J mice in a Nrf2-dependent manner. Infiltrating macrophages (CD11b^hi^ CD11c^lo^, % CD64+) in the lungs or spleens of A/J mice challenged with vinyl carbamate and treated with CDDO-Me as described in Figure 4 were detected by flow cytometry (**A**). Mean fluorescence intensity (MFI) of CD206 on lung-infiltrating macrophages (**B**) or spleen macrophages (**C**). (**D**) Immunofluorescent staining of CD206 in lung tumors. Scale bar = 60 microns. (**E**) Quantification of (**D**). (**A**–**C**) represent percentages of the parental gated population. Representative of n = 11–24 (**A**–**C**) or n = 3 (**D**,**E**) mice. Unpaired T test (**A**–**C**) or two-way ANOVA followed by Tukey HSD (**D**,**E**). ns = not significant, ** *p* < 0.01, **** *p* < 0.0001.

For immunophenotyping of mice with lung tumors, lungs and spleens of A/J mice treated as described in Figure 4 were harvested at necropsy, digested as previously described [59], and stained using an optimized flow panel [47]. There was a ~20% increase (*p* < 0.0001) in infiltrating (CD11b^hi^ CD11c^lo^) [60] macrophages in the lungs of WT mice treated with CDDO-Me but not in the lungs of Nrf2 KO mice treated with CDDO-Me (Figure 5A). Importantly, these infiltrating macrophages expressed significantly (*p* < 0.0001) lower surface levels of the tumor-promoting macrophage marker CD206, as measured by mean fluorescence intensity (MFI). No change in CD206 expression was observed on the surface of macrophages infiltrating the lungs of Nrf2 KO mice (Figure 5B). Notably, no changes were detected in alveolar macrophages (Figures S5A and S6A). When the groups were analyzed separately by sex, similar trends in macrophage infiltration and CD206 expression were observed in the lungs of female (Figure S5A) and male (Figure S6A) WT mice treated with CDDO-Me. However, the increased macrophage infiltration and decreased CD206 expression only reached statistical significance in female mice (Figure S5A). In contrast to the lungs, treatment with CDDO-Me significantly (*p* < 0.0001) increased CD206 MFI on macrophages in the spleens (Figure 5C) of both female (*p* < 0.0001) (Figure S5B) and male (*p* < 0.05) (Figure S6B) mice. The level of CD206 expression was also higher (*p* < 0.05) in WT female mice treated with CDDO-Me compared with WT male mice treated with CDDO-Me (Figure S7A). 

Subtle sex-dependent differences in T cell populations were also detected. Specifically, female WT mice on control diet had fewer (*p* < 0.05) CD4+ T cells in their spleens compared to WT male mice on control diet. This difference was, however, absent in WT male and female mice fed CDDO-Me diet (Figure S7B). Additionally, the CD107a MFI on CD25+ CD8+ T cells in the spleen was lower (*p* < 0.01) in female vs. male WT mice treated with CDDO-Me (Figure S7C). These changes in spleen macrophages and T cells were not observed in the spleens of Nrf2 KO mice, regardless of sex or treatment (Figure 5C, Figures S5B, S6B and S7C). 

Because a ~50% decrease in the expression of CD206 on infiltrating macrophages was observed in WT mice and because histological organization is lost in analysis by flow cytometry, additional lung sections were immunostained for CD206. The number of CD206-positive cells were lower (*p* < 0.01) around the periphery of tumors in the lungs of WT but not Nrf2 KO mice treated with CDDO-Me (Figure 5D,E). The proximity of these CD206+ cells to tumors suggests a direct interaction, which could exacerbate tumor progression by suppressing anti-tumor immunity [61].

## 4. Discussion

Because of the dense macrophage population in the lung tumor microenvironment and the phenotypic plasticity of these cells, targeting macrophage polarization is a promising strategy for novel lung cancer therapies [11,62,63,64]. The consequences of Nrf2 activation in immune cells including macrophages is highly context-dependent, and although Nrf2 activation in macrophages that have not been exposed to the cancer secretome has anti-inflammatory outcomes [30,65,66], the effects of Nrf2 activation on macrophages within the specific context of lung cancer has not been sufficiently investigated. We hypothesized that because pro-inflammatory macrophages rely on cytoprotective mechanisms like Nrf2 to maintain their anti-tumor phenotype [20,67], Nrf2 activation in macrophages could repolarize them to an anti-tumor phenotype within the TME and reduce lung tumor burden. The potential use of NRF2 pathway modulators for cancer prevention or treatment remains a controversial topic and the safety of NRF2 activating drugs for non-cancer diseases has been questioned due to the potential effects of NRF2 activation in tumor cells, namely drug resistance and increased survival [24]. Few studies to date, however, have directly investigated the effects of NRF2 activation on other cells within the TME.

Increases in pro-inflammatory (TNFα, IL-6, and MHC-II) and decreases in pro-tumor (VEGF, CCL2, and CD206) markers in WT tumor-educated BMDMs treated with 100 nM CDDO-Me suggest beneficial polarization of macrophages in vitro. Importantly, similar trends were observed at the transcriptional level by using conditioned media from two different mouse lung cancer cell lines, suggesting that this phenomenon is unlikely to be tumor cell line-specific and could be applied to a wider range of heterogenous lung tumors. These data were in striking contrast to the effects of Nrf2 activation in classically activated macrophages, as CDDO-Me decreased the anti-inflammatory cytokines TNFα and IL-6 in control BMDMs stimulated with LPS or IFN-γ. These results support previous findings that Nrf2 activation in macrophages has anti-inflammatory effects in non-cancer settings such as autoimmune diseases [30,65,66] but can have opposite effects within tumors [68,69]. 

BMDM experiments were analyzed at the transcriptional, protein, and cell surface marker levels, and similar phenotypic trends were observed in all three. Interestingly, increases in VEGF and CCL2 mRNA were either dampened or absent in Nrf2 KO BMDMs cultured in conditioned media from murine lung cancer cells (Figure 2B and Figure S1B). However, VEGF and CCL2 protein were both increased by conditioned media to a similar magnitude in WT and Nrf2 KO BMDMs (Figure 3A). Because the increase in these markers in response to conditioned media is undetectable on the transcriptional level at the 24 h time point, there may be a difference in transcriptional kinetics between Nrf2 WT and KO BMDMs. These results showcase that while macrophages within the TME are more frequently skewed toward the M2 phenotype, conventional polarization alone with cytokines (LPS, IFN-γ, IL-4, etc.) does not adequately model the behavior of tumor-associated macrophages. 

Our studies confirmed the previously observed anti-tumor effects of CDDO-Me [38,39] in lung cancer. However, the Nrf2 dependency for these striking anti-tumor effects had not previously been studied. Consequently, a whole-body Nrf2 KO mouse was generated on the A/J background, and the lack of response in Nrf2 KO mice confirms the requirement of Nrf2 for the efficacy of CDDO-Me in this model. Interestingly, tumor burden was markedly higher in the Nrf2 KO mice compared to WT mice. This observation is consistent with previously reported differences in tumor burden between WT and Nrf2 KO mice on the Balb/c background [33] and contributes additional evidence to the importance of Nrf2 in protecting cells in the lung from malignant transformation. 

However, disparate effects on tumor burden following Nrf2 KO were observed in other mouse models. While Nrf2 initially protected ICR/CD-1 mice from urethane-induced lung carcinogenesis, tumor burden was higher in WT animals compared to Nrf2 KO animals by 16 weeks after initiation [70]. In stark contrast to our results, Bauer et al. found that Nrf2 deletion reduced urethane-induced lung tumor burden in Balb/cCR mice [71]. There are a variety of factors which may explain these discrepancies, including the use of different carcinogens to induce tumorigenesis. While urethane is more commonly used than vinyl carbamate, this method of initiation produces adenomas which are less aggressive, less invasive, and slower growing than the adenocarcinomas induced by the active metabolite of urethane, vinyl carbamate [39]. Additionally, mouse strains with varying genetic backgrounds differ in their susceptibility to carcinogen-induced tumorigenesis, and immune cell activity also varies between different strains [72,73]. 

The majority of studies using the A/J mouse model have been done in female mice, so sex differences in this model have not been well characterized. Male mice appeared to be more sensitive than female mice to tumor initiation with vinyl carbamate in both WT and Nrf2 KO groups (Table S2) and thus had worse overall tumor burden and hampered anti-tumor responses. There were no statistically significant differences between the tumor grades of male and female mice within treatment groups (Table S3). These data suggest that while male A/J mice may have a higher tumor burden compared to female A/J mice after being challenged with vinyl carbamate, these differences in sensitivity do not alter the histopathology of the tumors, at least by 16 weeks after initiation. 

The lung tumor immune microenvironment had not been previously characterized in A/J mice treated with CDDO-Me, and macrophages were the most altered cell type within this compartment (Figure 5). Although increased infiltration of macrophages is often correlated with worse overall prognosis in lung cancer [74,75,76], an important distinction is that these infiltrating cells are mostly M2 tumor-promoting macrophages which continue to increase as tumor stage progresses [8]. Increased infiltration of M1 anti-tumor macrophages correlates with increased patient survival [77,78] and the decreased intensity of CD206 (a marker of M2 macrophages) staining on these infiltrating macrophages reveals that fewer of these cells were likely to promote tumor growth. These results were confirmed by immunofluorescent staining, which also showed the proximity of CD206-staining cells to tumors within the lungs. CD206 staining intensity on spleen macrophages, however, was increased with CDDO-Me in WT mice. These data are consistent with previous studies reporting that Nrf2 activation has anti-inflammatory effects in a non-cancer context [30,65,66], as A/J mice do not develop tumors in the spleen [79]. These opposing effects in different organs, with or without the presence of malignant lesions or the tumor secretome, demonstrate the range of physiological outcomes following Nrf2 activation. They also confirm our in vitro observations and the differences between macrophage polarization when induced by cytokines vs. conditioned media from lung cancer cells. 

While the dual role of Nrf2 in normal cells and established cancer cells has been well-characterized [24], less is known about the effects of Nrf2 during carcinogenesis. As cells acquire oncogenic mutations and undergo malignant transformation, Nrf2 activity in cancer cells increases [80,81,82]. This is in part due to the mitochondrial hyperactivity and subsequent ROS elevation in rapidly proliferating cells [83,84]. Therefore, basal Nrf2 activity in cancer cells is elevated compared to other cells in the immune microenvironment regardless of Nrf2 mutational status. These cells, including macrophages, may be more sensitive to Nrf2 activation with CDDO-Me comparatively, and therefore treatment would likely lead to clinical benefit in lung cancer patients as suggested by the results of our in vivo studies. In the vinyl carbamate-induced model used in our studies, treatment began two weeks post tumor initiation. In early lung cancer stages, anti-tumor polarized M1 macrophages are the dominant macrophage subtype [16], and these are gradually replaced with tumor-promoting macrophages as the tumor stage progresses. Since M1 macrophages rely on cytoprotective mechanisms like Nrf2 activation to maintain their anti-tumor phenotype [20], it is not entirely surprising that treatment with the Nrf2 activator CDDO-Me during early stages alters macrophage polarization and has anti-tumor effects in vivo. However, elevated Nrf2 activity can be detected in lung cancer cells regardless of Nrf2 mutation status [26,27]. The results described herein suggest that treatment with CDDO-Me polarizes macrophages to an anti-tumor phenotype and may therefore have beneficial anti-tumor effects even in late stages of lung cancer. Additional preclinical studies are currently underway to answer this important question.

## Figures and Tables

**Table 1:** Nrf2 KO exacerbates vinyl carbamate-induced lung tumorigenesis and CDDO-Me decreases tumor number, size, burden, and histopathological grade of tumors in WT mice. WT and Nrf2 KO mice were challenged with vinyl carbamate and treated as described in Figure 4. Two-way ANOVA (raw values) or z test (proportional values). * *p* < 0.05 vs. WT Control; ** *p* < 0.01 vs. WT Control; *** *p* < 0.001 vs. WT Control; **** *p* < 0.0001 vs. WT Control; ^#^ *p* < 0.05 vs. WT CDDO-Me; ^##^ *p* < 0.01 vs. WT CDDO-Me; ^###^ *p* < 0.001 vs. WT CDDO-Me; ^####^ *p* < 0.0001 vs. WT CDDO-Me.

	WT Control	WT CDDO-Me 50 mg/kg	Nrf2 KO Control	Nrf2 KO CDDO-Me 50 mg/kg
**Surface tumors**	618	106	1280	1289
Mice per group	23	24	23	24
Average # of tumors per mouse (% WT control)	26.87 ± 1.9 (100%)	4.42 ± 0.9 (16.4%) ****	55.65 ± 2.5 (207%) **** ^####^	53.71 ± 2.3 (200%) **** ^####^
**Tumor number, size, and burden**				
Number of slides per group	46	48	46	48
Average # of tumors per slide (% WT control)	2.52 ± 0.3 (100%)	0.96 ± 0.1 (38%) **	6.22 ± 0.4 (247%) **** ^####^	6.25 ± 0.5 (248%) **** ^####^
Average tumor size (mm^3^) per slide (% WT control)	0.26 ± 0.07 (100%)	0.03 ± 0.007 (13%) ***	0.49 ± 0.15 (189%) *** ^###^	0.34 ± 0.10 (131%) *** ^###^
Average tumor burden (mm^3^) per slide (% WT control)	0.65 ± 0.1 (100%)	0.03 ± 0.007 (5%) ***	3.02 ± 0.04 (467%) *** ^###^	2.10 ± 0.3 (324%) *** ^###^
**Tumor histopathology**				
Total # low grade (% total)	24 (21%)	21 (46%) *	46 (16%) ^##^	51 (17%) ^##^
Total # medium grade (% total)	44 (38%)	17 (37%)	111 (39%)	139 (46%)
Total # high grade (% total)	48 (41%)	8 (17%) *	129 (45%) ^##^	111 (37%) ^#^

## Data Availability

Any relevant data not presented in this study or as supplementary material are available by request from the corresponding author.

## Supplementary Materials

The following supporting information can be downloaded at: https://www.mdpi.com/article/10.3390/antiox12010116/s1.
