# Supplementary material for: The Triterpenoid CDDO-Methyl Ester Redirects Macrophage Polarization and Reduces Lung Tumor Burden in a Nrf2-Dependent Manner
Source: Antioxidants (Basel). 2023 Jan 3;12(1):116. doi: 10.3390/antiox12010116 (PMC9854457; doi:10.3390/antiox12010116)
Supplement: Supplementary file 1 [file antioxidants-12-00116-s001.zip › antioxidants-2126017-supplementary.pdf]

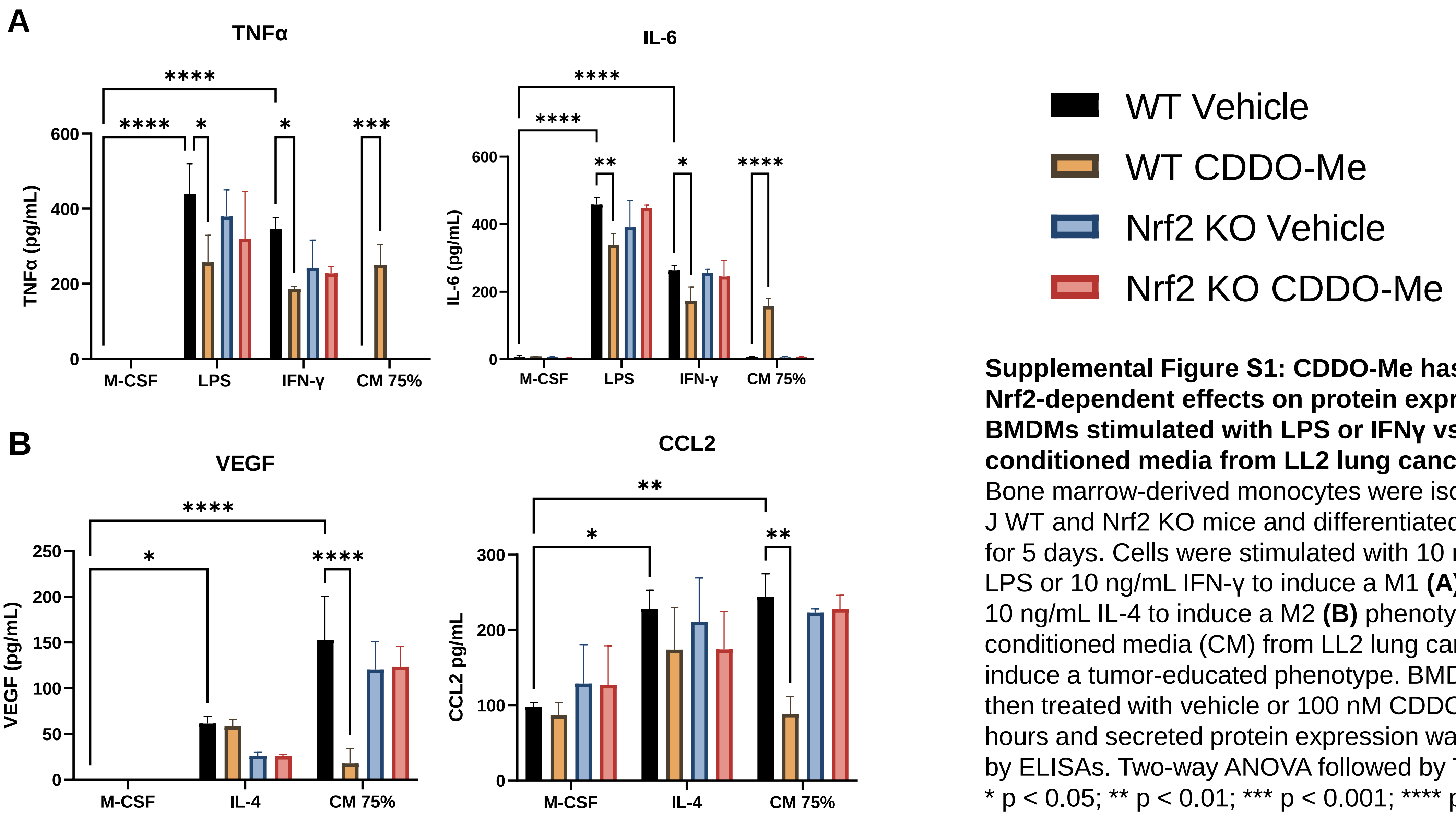

Supplemental Figure S1: CDDO-Me has opposing Nrf2-dependent effects on protein expression in BMDMs stimulated with LPS or IFNγ vs. conditioned media from LL2 lung cancer cells. Bone marrow-derived monocytes were isolated from A/J WT and Nrf2 KO mice and differentiated with M-CSF for 5 days. Cells were stimulated with 10 ng/mL LPS or 10 ng/mL IFN-γ to induce a M1 **(A)** phenotype, 10 ng/mL IL-4 to induce a M2 **(B)** phenotype, or conditioned media (CM) from LL2 lung cancer cells to induce a tumor-educated phenotype. BMDMs were then treated with vehicle or 100 nM CDDO-Me for 24 hours and secreted protein expression was analyzed by ELISAs. Two-way ANOVA followed by Tukey HSD. * $p < 0.05$; ** $p < 0.01$; *** $p < 0.001$; **** $p < 0.0001$

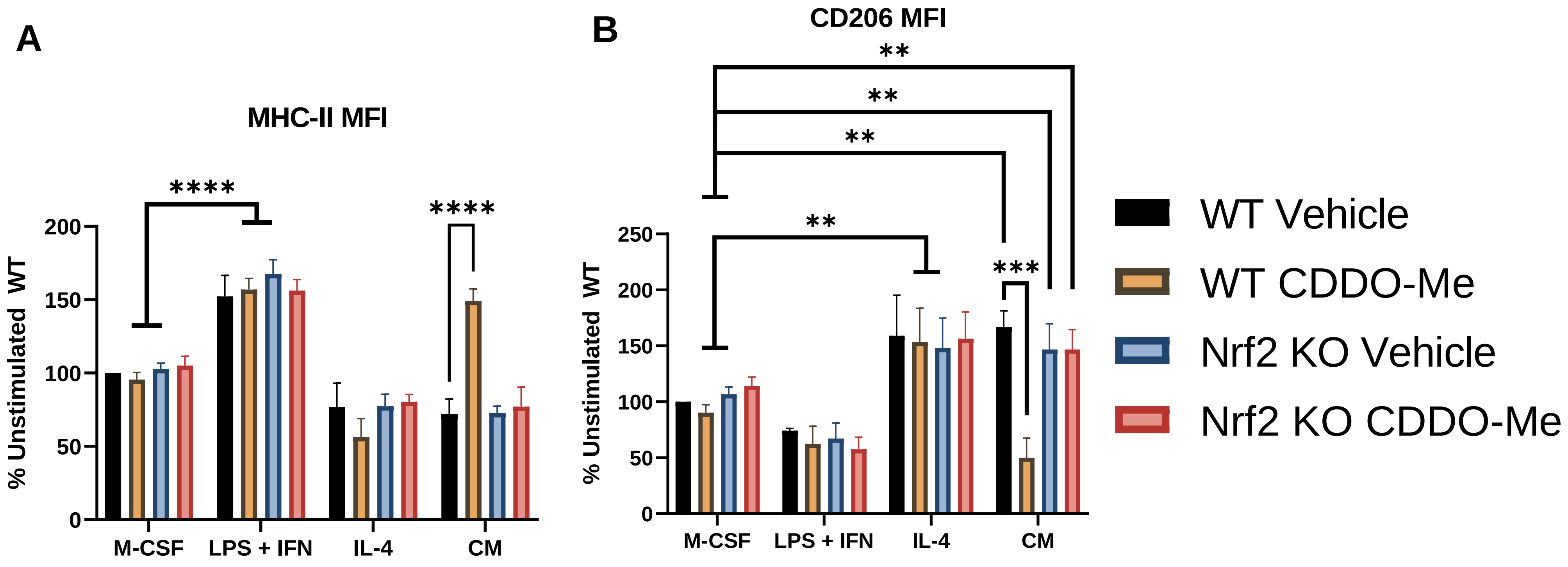

Supplemental Figure S2: CDDO-Me changes phenotypic surface marker expression on tumor-educated BMDMs in a Nrf2-dependent manner. BMDMs were isolated as described in Supp. Fig. 1 and treated with 100 nM CDDO-Me for 48 hrs. Surface marker expression was analyzed by flow cytometry. Mean fluorescence intensity (MFI) of MHC-II **(A)** and CD206 **(B).** Two-way ANOVA followed by Tukey HSD. ** $p < 0.01$; *** $p < 0.001$; **** $p < 0.0001$

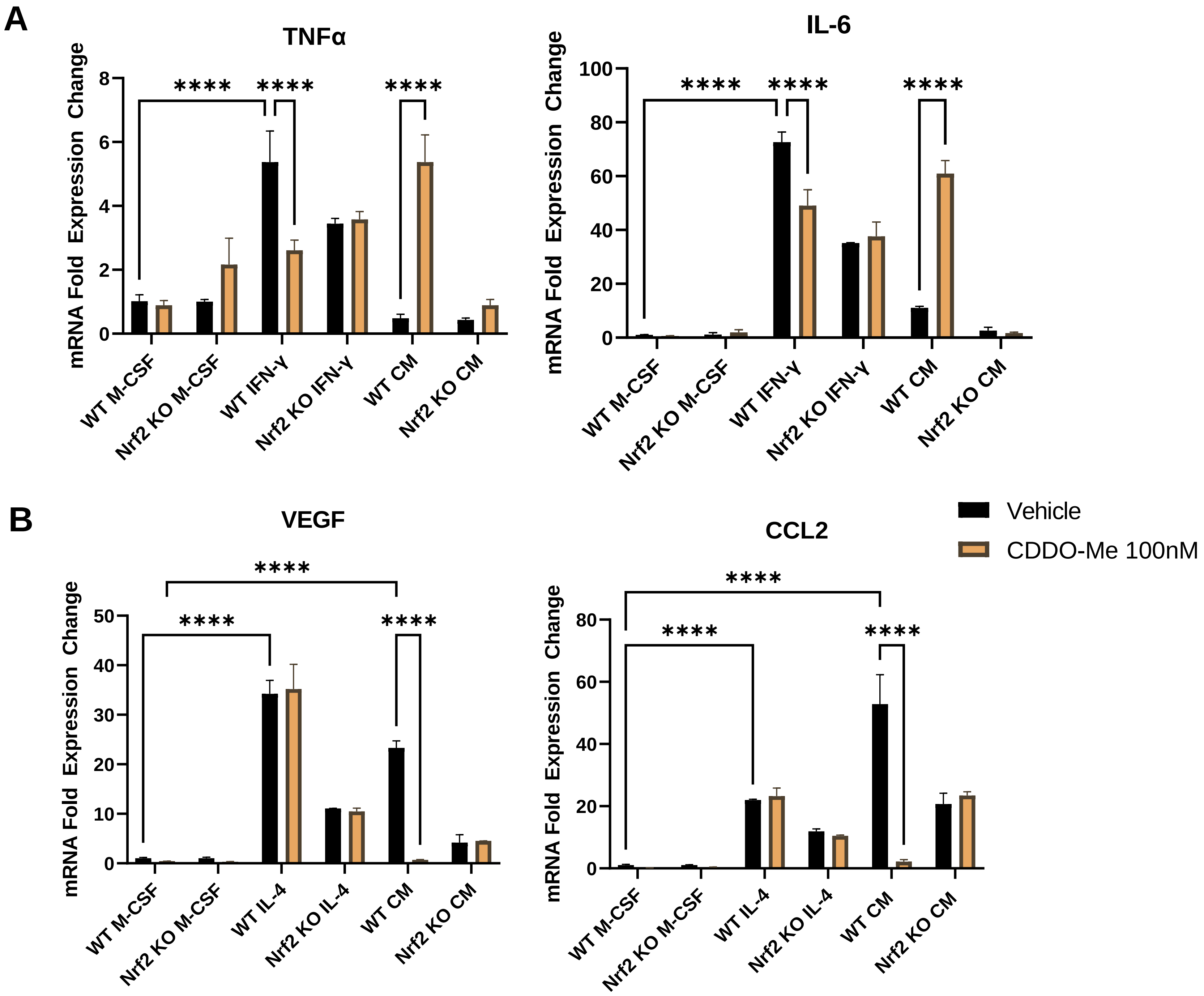

Supplemental Figure S3: CDDO-Me has opposing Nrf2-dependent effects in BMDMs stimulated with LPS or IFNγ vs. conditioned media from VC-1 lung cancer cells. Bone marrow-derived monocytes were isolated from A/J WT and Nrf2 KO mice and differentiated with M-CSF for 5 days. Bone marrow-derived macrophages were stimulated with 10 ng/mL LPS or 10 ng/mL IFN-γ to induce a M1 **(A)** phenotype, 10 ng/mL IL-4 to induce a M2 **(B)** phenotype, or conditioned media (CM) from VC-1 lung cancer cells to induce a tumor-educated (**A, B**) phenotype. BMDMs were then treated with vehicle (black bars) or 100 nM CDDO-Me (orange bars) for 24 hours and mRNA expression was analyzed by qPCR. Two-way ANOVA followed by Tukey HSD. **** $p < 0.0001$.

Target	Source	Forward	Reverse
GAPDH	IDT	5’-CATCACTGCCACCCAGAAGACTG-3’	5’-ATGCCAGTGAGCTTCCCGTTCAG-3’
TNFα	IDT	5’-AAGCCTGTAGCCCACGTCGTA-3’	5’-GGCACCACTAGTTGGTTGTCTTTG-3’
IL-6	IDT	5’-CACGGCCCTCCCTACTTCAC-3’	5’-TGCAAGTGCATCATCGGTGT-3’
VEGF	IDT	5’-CTCACCAAAGCCAGCACATA-3’	5’-AATGCTTTCTCCGCTCTGAA-3’
NQO1	IDT	5’-AATGGGCCAGTACAATCAGG-3’	5’-CCAGCCCTAAGGATCTCTCC-3’
HO-1	IDT	5’-CCTTCCCGAACATCGACAGCC-3’	5’-GCAGCTCCTCAAACAGCTCAA-3’
**Target**	**Source**	**Catalog #**	
CCL2	Qiagen	PPM03151G-200	

Supplemental Table S1: Primer sequences used in qPCR experiments.

	**Male**	**Female**
**WT Vehicle**	27.4 ± 1.2	21.9 ± 0.9
**WT CDDO-Me 50mg/kg**	25.9 ± 0.9	19.9 ± 0.9
**Nrf2 KO Vehicle**	25.9 ± 0.5	21.4 ± 0.5
**Nrf2 KO CDDO-Me 50mg/kg**	25.8 ± 0.4	21.2 ± 0.6

Supplemental Table S2: Final weights of WT and Nrf2 KO A/J mice. Average weight (g) ± standard error.

	F WT Control	M WT Control	F WT Me	M WT Me
**Surface tumors**	321	297	47	59
Mice per group	12	11	12	12
Avg # tumors per mouse (% WT control)	26.8±3.0 (100%)	27±1.8 (100%)	3.9±0.6 (14.6%)	4.9±0.8 (18.2%)
**Tumor number size & burden**				
Avg # tumors/slide (% WT control)	2.96±0.4 (100%)	2.05±0.3 (100%)	0.63±0.1 (21.3%)	1.29±0.3 (63.1%)* vs. F WT Me
Avg tumor size (mm³)/slide (% WT control)	0.21±0.08 (100%)	0.33±0.1 (100%)	0.02±0.005 (11.7%)	0.04±0.01 (10.9%)
Avg tumor burden (mm³)/slide (% WT control)	0.61±0.1 (100%)	0.68±0.2 (100%)	0.02±0.004 (2.51%)	0.05±0.01 (6.9%)* vs. F WT Me
**Tumor histopa**thology				
Total # low grade (% total)	19 (27%)	5 (11%)	7 (47%)	14 (45%)
Total # medium grade (% total)	28 (39%)	16 (36%)	5 (33%)	12 (39%)
Total # high grade (% total)	24 (34%)	24 (53%)	3 (20%)	5 (16%)

	F KO Control	M KO Control	F KO Me	M KO Me
**Surface tumors**	608	672	662	627
Mice per group	11	12	12	12
Avg # tumors per mouse (% WT control)	55.3±2.7 (206.6%)	56±3.1 (207.4%)	55.2±3.1 (206.2%)	52.3±1.6 (193.5%)
**Tumor number size & burden**				
Avg # tumors/slide (% WT control)	6.36±0.5 (215.1%)	6.08±0.5 (297.4%)	6.5±0.8 (219.7%)	6.0±0.6 (293.3%)
Avg tumor size (mm³)/slide (% WT control)	0.3±0.1 (144.4%)	0.67±0.2 (198.8%)	0.3±0.1 (142.5%)	0.38±0.2 (113.5%)
Avg tumor burden (mm³)/slide (% WT control)	1.91±0.3 (310.6%)	4.05±0.6 (591.4%)* vs. F KO V	1.92±0.3 (313.1%)	2.28±0.3 (333%)
**Tumor histopa**thology				
Total # low grade (% total)	30 (21%)	16 (11%)	28 (18%)	23 (16%)
Total # medium grade (% total)	58 (41%)	53 (36%)	87 (56%)	52 (36%)
Total # high grade (% total)	52 (37%)	77 (53%)	41 (26%)	70 (49%)

Supplemental Table S3: Sex differences within treatment groups of WT and Nrf2 KO A/J mice. Separation of data shown in Table 1 by sex. Only statistically significant sex-dependent differences are listed. Unpaired T test. * $p < 0.05$

	WT Control	WT CDDO-Me 12.5mg/kg	Nrf2 KO Control	Nrf2 KO CDDO-Me 12.5mg/kg
**Total surface tumors**	**281**	**149**	**252**	**263**
Mice per group	**11**	**10**	**5**	**5**
Avg per mouse (% WT Control)	**25.5 ± 1.8 (100%)**	**14.9 ± 0.6 (58.3%) ***	**50.4 ± 2.6 (197.3%) *******	**52.6 ± 3.1 (205.9%) *******
**Tumor #, size, & burden**				
# of slides/group	**22**	**20**	**10**	**10**
Avg # of tumors per slide (% WT control)	**2.6 ± 0.3(100%)**	**0.96 ± 0.3 (38%) ***	**6.22 ± 0.5 (246.6%) * #**	**6.25 ± 0.8 (247.8%) * #**
Avg tumor size (mm^3) (% WT control)	**0.23 ± 0.07 (100%)**	**0.1 ± 0.03 (42.3%) ***	**0.49 ± 0.17 (207.2%) ##**	**0.56 ± 0.21 (236.8%) *** ###**
Avg tumor burden (mm^3) (% WT control)	**0.61 ± 0.11 (100%)**	**0.19 ± 0.04 (30.9%) ***	**1.2 ± 0.28 (197.4%) * ##**	**1.8 ± 0.39 (301.1%) ** ##**
**Tumor histopathology**				
Total # low grade (% total)	**6 (10.5%)**	**8 (21.6%)**	**5 (18.5%)**	**2 (6.1%)**
Total # med grade (% total)	**27 (47.4%)**	**21 (56.8%)**	**10 (37%)**	**13 (39.4%)**
Total # high grade (% total)	**26 (45.6%)**	**12 (32.4%)**	**12 (44.4%)**	**18 (54.5%)**

Supplemental Table S4: Tumor numbers, sizes, burden, and histopathology of WT and Nrf2 KO A/J mice treated with vehicle or 12.5 mg/kg CDDO-Me. Tumors were initiated as described and mice treated with 12.5mg/kg CDDO-Me. Two-way ANOVA followed by Tukey HSD for multiple comparisons of tumor number, size, and burden; z-test for proportions of histopathological grades. * $p < 0.05$ vs. WT control; ** $p < 0.01$ vs. WT control; *** $p < 0.001$ vs. WT control; **** $p < 0.0001$ vs. WT control; # $p < 0.05$ vs. WT CDDO-Me 12.5 mg/kg; ## $p < 0.01$ vs. WT CDDO-Me 12.5mg/kg; ### $p < 0.001$ vs. WT CDDO-Me 12.5mg/kg.

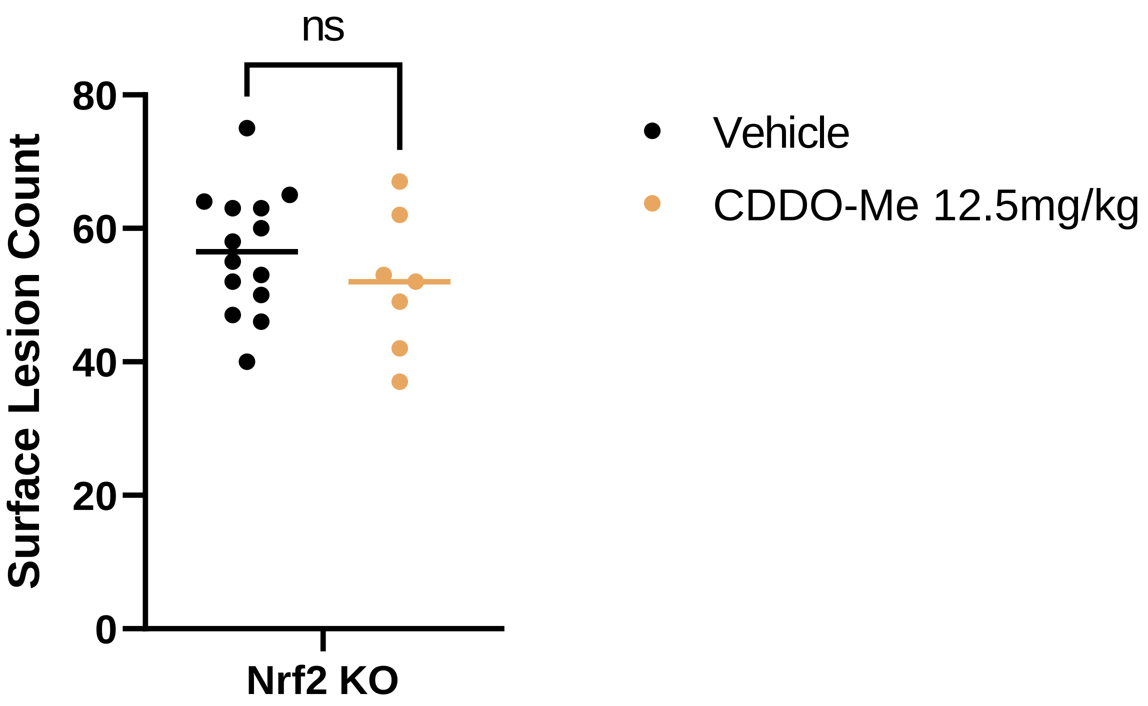

Supplemental Figure S4: A low dose of CDDO-Me does not change surface tumor counts in A/J Nrf2 KO mice. Surface tumor quantification of Nrf2 KO mice treated with vehicle control or CDDO-Me (12.5 mg/kg). Unpaired T test. ns = not significant.

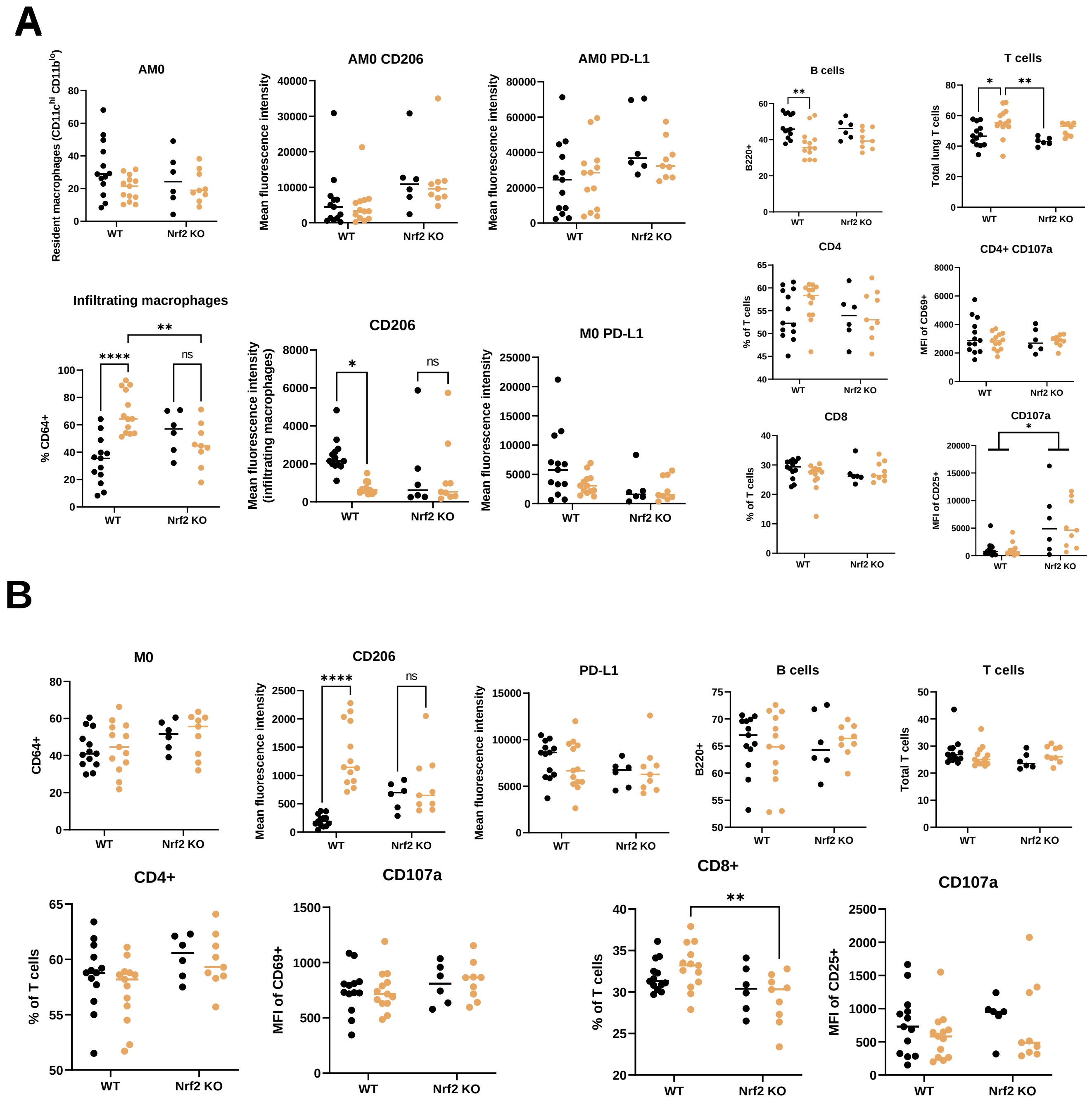

Supplemental Figure S5: Immune cell populations in the lungs (A) and spleens (B) of female A/J mice. Female A/J WT and Nrf2 KO mice were challenged with vinyl carbamate and fed CDDO-Me in diet for 16 weeks. Immune cell populations were analyzed by flow cytometry. 2-way ANOVA followed by Tukey HSD. ns = not significant; * $p < 0.05$; ** $p < 0.01$; *** $p < 0.001$; **** $p < 0.0001$

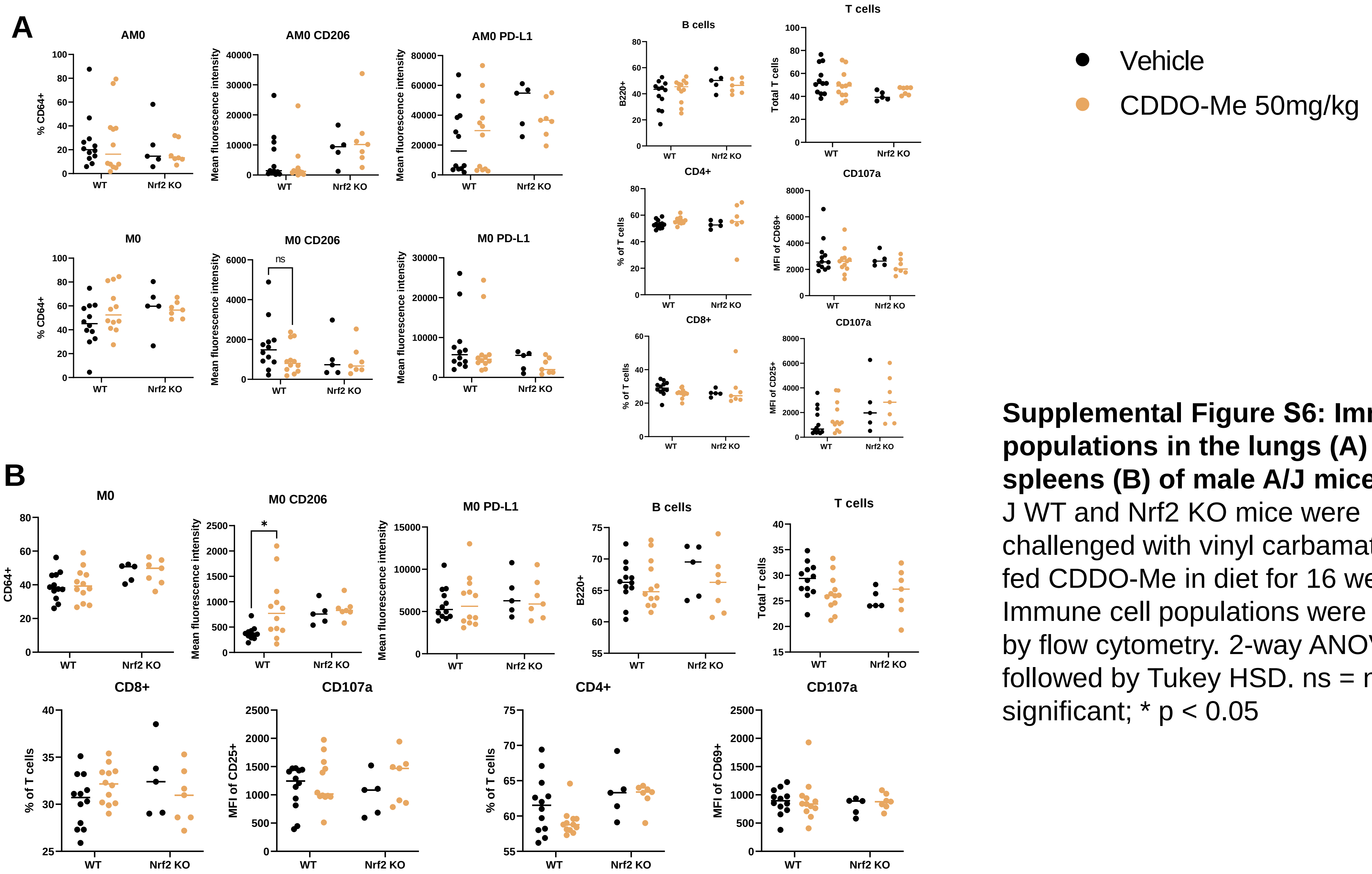

Supplemental Figure S6: Immune cell populations in the lungs (A) and spleens (B) of male A/J mice. Male A/J WT and Nrf2 KO mice were challenged with vinyl carbamate and fed CDDO-Me in diet for 16 weeks. Immune cell populations were analyzed by flow cytometry. 2-way ANOVA followed by Tukey HSD. ns = not significant; * $p < 0.05$

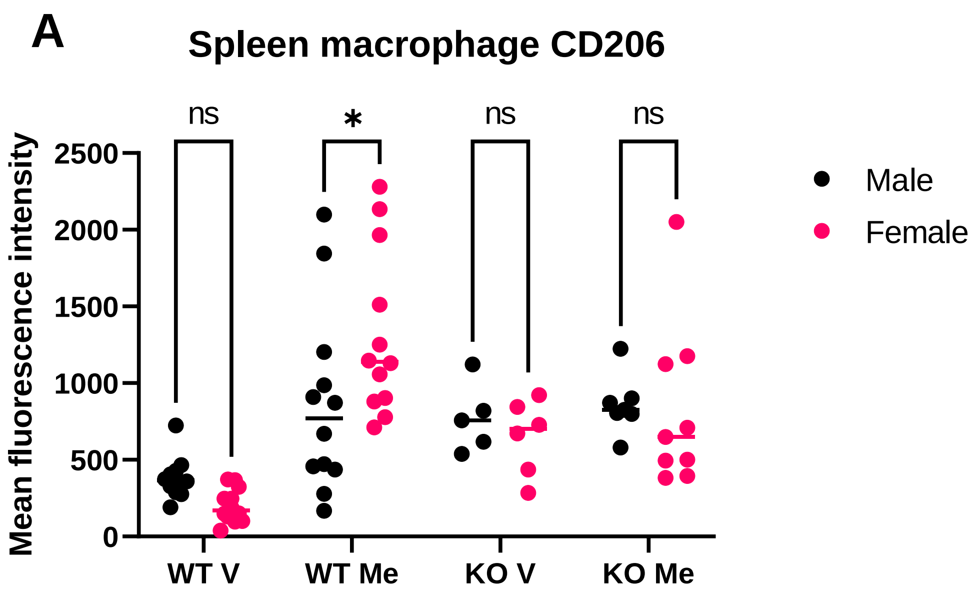

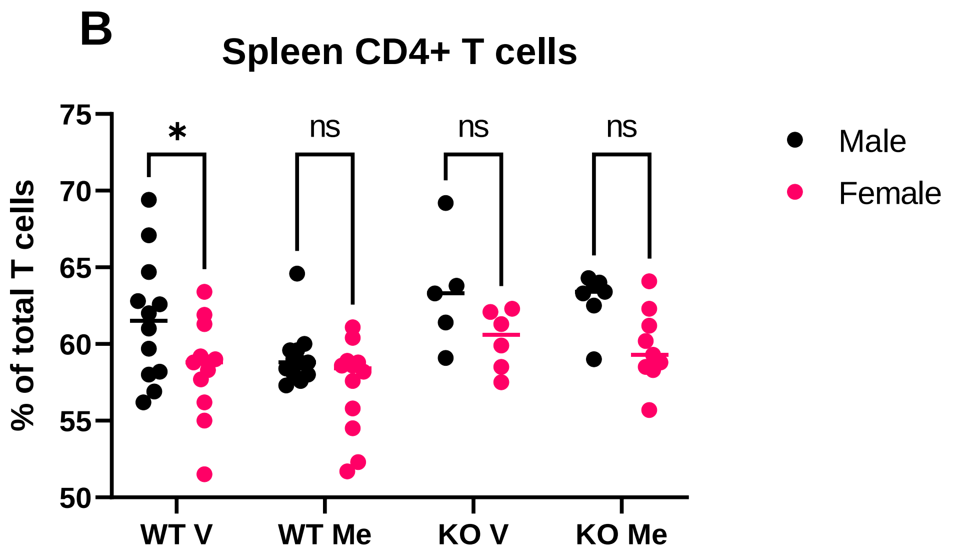

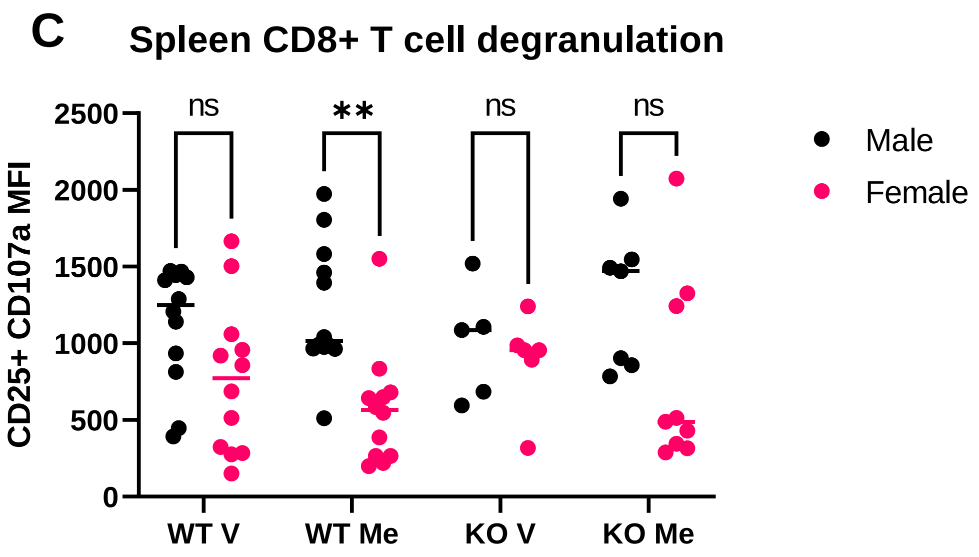

Supplemental Figure S7: Sex differences in immune cell populations in A/J mice. A/J WT and Nrf2 KO mice were challenged with vinyl carbamate and fed CDDO-Me in diet for 16 weeks. Immune cell populations were analyzed by flow cytometry. **(A)** CD206 MFI on spleen macrophages. **(B)** Total CD4+ T cells in the spleen. **(C)** CD107a MFI on CD25+ cytotoxic (CD8+) T cells in the spleen. 2-way ANOVA followed by Tukey HSD. ns = not significant; * $p < 0.05$; ** $p < 0.01$
